# Nutritional status and its association with quality of life among people living with HIV attending public anti-retroviral therapy sites of Kathmandu Valley, Nepal

**DOI:** 10.1186/s12981-015-0056-9

**Published:** 2015-05-03

**Authors:** Rajshree Thapa, Archana Amatya, Durga Prasad Pahari, Kiran Bam, M Sophia Newman

**Affiliations:** Institute of Medicine, Maharajgunj Medical Campus, Tribhuwan University, Kathmandu, Nepal; James P Grant School of Public Health, BRAC University, Dhaka, Bangladesh; Independent Scholar, Chicago, IL USA

**Keywords:** Nutritional status, Quality of life (QoL), People living with HIV (PLHIV), Nepal, Food security

## Abstract

**Background:**

Little evidence exists on the connections between nutrition, diet intake, and quality of life (QoL) among people living with HIV (PLHIV). The study aimed to estimate the prevalence of under-nutrition among PLHIV in Nepal, and identify risk factors and assess correlations with PLHIVs’ QoL and nutritional status.

**Methods:**

This quantitative cross-sectional study used Body Mass Index (BMI) as an indicator for nutritional status, and additional information on opportunistic infections (OIs), CD4 count, and World Health Organization (WHO) clinical staging was collected from medical records. Participants were asked to complete surveys on food security and QoL. Descriptive analysis was used to estimate the prevalence of under nutrition. To assess associations between nutrition status and independent variables, bivariate and multivariate analysis was completed. Spearman’s rank correlation test was used to assess the association between nutritional status and QoL.

**Results:**

One in five PLHIVs was found to be under nourished (BMI <18.5 kg/m^2^). Illiteracy, residence in care homes, CD4 cells count <350 cells/mm^3^, OIs, and illness at WHO clinical stages III and IV were found to be significant predictors of under nutrition. BMI was significantly correlated with three domains of QoL (psychological, social and environmental).

**Conclusion:**

Nutrition interventions should form an integral part of HIV care programs. Understanding the presence of OI, decline in CD4 count, and advancing WHO clinical stages as risk factors can be helpful in preventing under nutrition from developing. Longitudinal research is necessary to further explicate associations between nutritional status and QoL.

## Introduction

Per Joint United Nations Programme on HIV and AIDS (UNAIDS), the number of people newly infected with Human Immunodeficiency Virus (HIV) each year is continuing to decline in most parts of the world. The new infections declined by 38 percent from 3.4 million in 2001 to 2.1 million in 2013 [1]. Meanwhile, care is increasing. The percentage of people living with HIV (PLHIV) who are receiving antiretroviral therapy (ART) have increased from around 10 percent in 2006 to around 37 percent in 2013, with 12.9 million people receiving ART worldwide by the end of 2013 [1]. As a result, Acquired Immune Deficiency Syndrome (AIDS)-related deaths have fallen by 35% since 2005, when the highest number of deaths was recorded [2]. As a result of life-saving treatment, the number of PLHIV is rising in spite of falling rates of new infection. At the end of 2013, there were approximately 35 million PLHIV [2].

The role of HIV infection on nutrition was identified early in the epidemic [3]. Wasting is one of the most visible signs of malnutrition as patients progress from HIV to AIDS [3]. HIV was found to affect nutritional status by increasing energy requirements, reducing food intake, and adversely affecting nutrient absorption and metabolism [4]. Failing to meet nutritional needs may lead to decreased immunity and increased susceptibility to opportunistic infections (OIs), which can lead to further malnutrition. Additionally, nutrient intake can improve antiretroviral absorption and tolerance [5]. Receiving appropriate nutrition can help improve PLHIV’s quality of life (QoL) [6]. Poor nutritional status in PLHIV speeds the disease progression, increases morbidity, and reduces survival time [7]. For these reasons, nutritional support should be a fundamental part of a comprehensive response to HIV and AIDS [8]. The World Health Organization (WHO) recommends ensuring micronutrient needs are met by increasing access to a diversified diet, fortified foods, and micronutrient supplements, particularly in areas where micronutrient deficiencies are endemic [8]. However, these clinical issues remain common, despite improvements in the treatment and survival of PLHIV [7].

Similarly, food insecurity and HIV and AIDS are intertwined in a vicious cycle [9]. Food insecurity is the condition of not having physical or economic access to enough food to be productive and healthy. Each condition increases the vulnerability to and worsen the severity of the other [9]. Among PLHIV, food insecurity is associated with incomplete HIV-1 RNA suppression, CD4 decline, increased opportunistic infections, hospitalizations, and HIV-related mortality [10]. Good nutrition for PLHIV has been proven to increase resistance to infection, help PLHIV maintain weight, and improve QoL, drug compliance, and drug efficacy [10,11].

In a chronic disease like HIV and AIDS the QoL of the patients is also important [12]. The WHO defines QoL as “an individuals’ perception of their position in life in the context of their culture and value systems in which they live and in relation to their goals, standards, expectations and concerns” [13]. Health Related QoL (HRQoL) comprises the components of QoL that are directly related to health status. Studies have reported a strong association between HRQoL and socioeconomic characteristics [14,15] and health related indicators [16] in resource-poor settings. Various symptoms, including wasting, are known to potentially reduce QoL among PLHIV [17]. But the relationship between nutritional status and QoL are not yet clearly defined [10], and the investigation of the relationship between food security and HRQoL among PLHIV is just beginning to emerge [10,11,18]. In particular, there is a lack of clarity on the dimensions of food security critical for improving HRQoL for PLHIV. By the latest estimate (2013), the current prevalence of HIV is 0.23 percent among adults (aged 15-49 years) in Nepal [19]. A total of 40,720 people are estimated to be living with HIV [19]. There has been a large decline in new infections annually, from 8,039 in 2000 to 1,408 in 2013 [19]. Per the country’s Millennium Development Goal 2013 progress report, around 28.7 percent of PLHIV with advanced HIV infection are now receiving antiretroviral combination therapy, a figure that increased from 21 percent in 2010 [20].

A recent study from the Food and Agricultural Organization (FAO) shows that in Nepal, around 3.7 million people, representing 16.4 percent of the rural population of Nepal, are food insecure [21]. The number of these who are PLHIV is difficult to ascertain. In a food insecure area, assessing food security during clinical care for the PLHIV population is important. This study aims to identify nutritional status of PLHIV in Nepal, to identify how food security may impact nutritional status, and to clarify the relationship between nutritional status and HRQoL and PLHIV in Nepal.

## Methods

### Study design

This is a cross-sectional study using quantitative domain to assess the relationship between nutrition status and other variables.

### Study location and timeframe

We conducted this study at two public ART sites, Tribhuvan University Teaching Hospital (TUTH) and Sukraraj Tropical and Infectious Disease Control Hospital (STIDH). These were selected out of four public ART sites in Kathmandu Valley, Nepal, that provide ART. TUTH and STIDH were taken purposively based on the high number of PLHIVs registered at these two sites. Data was collected for six months, from July to December 2013.

### Study population and sampling technique

The study population was PLHIV who are accessing services from these health institutions. Inclusion criteria were a diagnosis of HIV at least six months prior to the study period, age 18 years or more, and ability to consent to the study. Both ART and pre-ART clients were included, and for those participants on ART, only those clients who have been taking ART for at least six months prior to the study period were included. As per the national ART guideline [22], those PLHIV in WHO stages III and IV or with CD4 cells count less than 350 cells/mm^3^ are enrolled on ART.

The exclusion criteria were PLHIV who are severely sick or unable to respond. In addition, pregnant women were excluded and those clients who had started ART within six months of the study were excluded. This last exclusion was to allow for accurate comparison of ART and pre-ART clients.

Other studies have established estimated malnutrition prevalence among PLHIV of 30% [23,24]. With a confidence interval of 95%, an allowable error of 5% and a 5% non-response rate, the total calculated sample size was 340. The sample was determined using formula by Kothari [25].

Clients on ART were identified via the ART and pre-ART registers maintained at the sites, and a random sampling technique was employed to select 340 prospective research participants. These 340 people were contacted for study recruitment. As PLHIV is recommended for the regular CD4 cells count monitoring, prospective participants were approached during their visit at health facility for ART or pre-ART services. At first contact, researchers briefly screened prospective participants to ensure that participants enrolled in the study were those who had been diagnosed with HIV for at least six months, and that those who were on ART, had been on the regimen for a minimum duration of six months.

### Ethics and consent

To accommodate the sensitivity of the issue and maintain participant confidentiality, verbal informed consent was taken from each participant before data collection. The informed consent process included a verbal explanation describing the purpose of the study, potential risks and benefits of participating, procedures for maintaining confidentiality, and the participants’ right to refuse to participate. Each participant was then given an opportunity to provide verbal consent, and this was recorded by the research team.

Acknowledging the sensitivity of the issue, researcher behavior that reinforced prevailing stigma and discrimination against PLHIVs was strictly prohibited. Sensitivity and appropriate language were included as topics in researcher preparation prior to data collection.

This informed consent followed the ethical norms and values stated in the National Ethical Guidelines for Health Research in Nepal (2001) [26]. Ethical approval was obtained from the Institutional Ethical Review Board (ERB) of the Institute of Medicine, Tribhuvan University.

### Data collection instruments

The study collected anthropometric data: a reference weight and height measured by standard methods, and a body mass index (BMI) calculated as a basic indicator of nutritional status. CD4 cell counts and WHO HIV and AIDS clinical staging data were taken from the health records of each participant. Each participant’s ART use status was also taken from client’s personal ART and pre-ART records and was confirmed at the time of recruitment. Clinical information such as the enrollment in ART, time since diagnosis, WHO clinical staging, and TB co-infection at any time after the diagnosis of HIV were recorded from individual clients records and triangulated with the ART and pre-ART registers maintained at the service facility.

Following this, each participant completed a questionnaire. The participant’s demographic information (age, literacy, educational level, marital status, caste, religion, and household status) and health behaviors (tobacco use and alcohol consumption) were included as self-report items on the first part of the questionnaire. To assess the QoL of the patients, the 26-item WHOQOL-HIV BREF [27] was used. This instrument has been utilized in many studies to determine the health related quality of life among PLHIV [28,29]. The instrument uses 26 items and measures QoL across four domains: physical well-being, psychological well-being, level of independence, and environment. The participant ranked their QoL on each item on a scale of one to five, where higher scores indicated greater QoL.

Household food insecurity was measured using Household Food Insecurity Access Scale (HFIAS) [30], a well-validated instrument used to measure household food insecurity in many countries [31-33]. It consists of nine questions on experiences of food insecurity, with possible responses of never, rarely, sometimes, or often. The highest score for HFIAS is 27; the higher the score, the greater the food insecurity.

The data collection tools were translated into the Nepali language and pre-tested with 10% of the sample size in the ART center at TUTH in Kathmandu. These samples were not included in the final analysis. Factual information obtained from these samples was cross-checked with ART and Pre-ART registers at the study sites. After the pretesting, various questions relating to co-infections were omitted, as only TB was found to have been recorded accurately. Similarly questions pertaining to fortified food supply from the ART center was also omitted as the supply of the fortified food was interrupted for more than six months during the time of study and very few study participants could actually recall of the receipt. Additionally, face validity was checked during the pretest and modifications were made to increase cultural competency in the question phrasing. Finally, the pre-testing process included discussion of stigma and discrimination with pre-testing participants, and their feedback was integrated into ensuring sensitive language and behavior was maintained in the data collection processes.

### Statistical analysis

Every completed questionnaire was reviewed at the end of each day to ensure its consistency and completeness. After data collection, 10 percent of the sample was randomly cross-checked for accuracy, and where errors were found, these were corrected. All recorded data was coded to facilitate data entry process. BMI was calculated from the height and weight using the standard formula: BMI = Weight (kg)/Height^2^ (m). The WHOQOL- HIV BREF [27] was used to produce a QoL profile derived from four domain scores denoting facets of an individual’s perceived QoL. The mean score of items within each domain is used to calculate the domain score. Mean scores are then multiplied by four in order to make domain scores comparable with the scores used in the WHOQOL-100, a commonly used scale. The instruments’ guidelines for checking and cleaning data and computing domain scores were rigorously followed.

Where more than 20% of data is missing from a questionnaire, the questionnaire was discarded. Where an item is missing, the mean of other items in the domain was substituted. Where more than two items are missing from the domain, the domain score was not calculated (with the exception of domain 3, where the domain could only be calculated if <1 item is missing). Finally, two items were examined separately: a question about an individual’s overall perception of QoL and a question about an individual’s overall perception of their health.

The calculation of the household food security score was based on the HFIAS version 3 [30]. The instrument had a top score of 27 points, where greater scores indicate more severe food insecurity. Four strata of food security were created based on the guideline (secure, mildly insecure, moderately insecure, and severely insecure). For the analysis purpose HFIA category 1 is termed as food secure and HFIA category 2 to 4 are categorized as food insecure. To assess associations between nutrition status and independent variables, bivariate and multivariate analysis was completed. Spearman’s rank correlation test was used to assess the association between nutritional status and QoL.

## Results

### Total participants

A total of 340 PLHIV were contacted for interviews in the two public ART sites in Kathmandu Valley; 120 were approached in TUTH and 220 were approached in STIDH. Of these, 15 people declined to participate. The response rate was therefore 95.58% (325/340). The major reasons for non- participation in TUTH was fear of breach of confidentiality (n = 6). Many of the clients who declined said they had not disclosed their status to anyone. In STIDH the major reason for non-participation was inability to commit the necessary amount of time (n =5), although other feared disclosure of their status or were disinterested in the study (n = 4).

Among the 325 interviews taken, 24 were not complete. Thus, 301 samples were taken for final analysis, for a final response rate of 88.56%. Of these, 101 participants from TUTH and 200 participants from STIDH completed the study.

### Population characteristics

The mean age of the population was 36.2 years with the standard deviation of 8.2 years. The majority of respondents (61.1%) were male. About one-third (30.2%) had completed primary level education or less, and around 77 percent were able to read and write. The majority were married (70.7%). A considerable percent were widows or widowers (8.6%). With respect to caste (the local equivalent of socioeconomic class); Janajatis (around 41%) accounted for the highest proportion of the PLHIVs seeking treatment at these sites, followed by Brahmins/Chettri (33%) and Dalits (18.6%). The majority were Hindus (70.8%), followed by Buddhists (15.9%), Christians (7%), and other faiths, mainly Kirat (3%) and Muslim (1.3%). Most resided with their family (87%), although 9% were living in group homes run by charitable organizations or in prison, and another 4% were living alone in rented homes. Background characteristics of the study participants are given in Table 1.

**Table 1:** **Socio demographic characteristics of study participants**

**Variables**	**N (%)**
**Treatment sites**	
TUTH	101(33.6%)
STIDH	200(66.4%)
**Age** (Mean ± SD )	36.2 ± 8.2
≤36 years	159(52.8%)
>36 years	142(47.2%)
**Gender**	
Male	184(61.1%)
Female	117(38.9%)
**Literacy status**	
Yes	233(77.4%)
No	68(22.6%)
**Educational level**	
Illiterate	68(22.6%)
Primary	91(30.2%)
Secondary	68(22.6%)
Higher secondary and above	74(24.6%)
**Marital status**	
Married	213(70.7%)
Unmarried	38(12.6%)
Divorced	12(4.0%)
Separated	12(4.0%)
Widower/Widowed	26(8.6%)
**Caste**	
Dalit	57(18.6%)
Terai Janajati	12(4.0%)
Janajati	123(40.9%)
Brahmin/Chettri	100(33.2%)
Other	9(3.0%)
**Place of residence**	
Residing with family	262(87.0%)
Residing with a care organization	27(9.0%)
Residing alone	12(4.0%)
**Religion**	
Hindu	219(70.8%)
Muslim	4(1.3%)
Buddhists	48(15.9%)
Christian	21(7.0%)
Others	9(3.0%)
**Occupation**	
Agriculture	54(17.9%)
Labor	23(7.6%)
Service	61(20.3%)
Self-business	46(15.3%)
Household work	61(20.3%)
No work	35(11.6%)
Others	21(7.0%)
**Smoking habits**	
Regular smokers	111(36.9%)
Non smokers	190(63.1%)
**Alcohol use**	
Regular alcohol user	98(22.6%)
Non alcoholic	203(67.4%)

### Nutritional status of PLHIV

To determine nutritional status, BMI was calculated. The standard cut-offs were used: <18.5 kg/m^2^ is underweight, 18.5-24.9 kg/m^2^ is normal, and greater than or equals to 25.0 kg/m^2^ is considered overweight. Out of the 301 samples analyzed, 60 participants had BMI less than 18.5 kg/m^2^. Thus 19.93% of the PLHIVs visiting the ART centers in Kathmandu valley were undernourished. Females were found to be more undernourished than the males; 17.4 percent of males and 23 percent of females had BMI less than 18.5 kg/m^2^. On the other hand, 29 PLHIV (9.7%) were found to be overweight. Details are presented in Table 2.

**Table 2:** **Nutritional status of PLHIV**

**BMI**	**N (%)**
BMI < 18.5 kg/m^2^ (underweight)	60 (19.9%)
BMI (18.5-24.4 kg/m^2^) (Normal weight)	212 (70.4%)
BMI (>24.4 kg/m^2^ (overweight)	29 (9.7%)
**Total**	301 (100%)

### Socioeconomic, behavioral factors, HIV related factors and nutrition status

The association between the various socioeconomic and HIV related variables and nutritional status was assessed through binary logistic regression. The statistical significance was tested at 95% confidence interval or p-value <0.05.

Among the demographic variables, age, gender, ethnicity, religion and occupation were found to have no significant association with nutritional status of the PLHIV. The mean age of the study participants was 36.2 ± 8.2, and the age of the study participants was not significantly associated with the nutritional status of the PLHIV.

Education status was found to be significantly associated with the nutritional status of PLHIV. Those PLHIV who were illiterate were almost 2.5 times more likely to be undernourished than those who were literate (Crude OR = 2.45; 95% C.I, 1.32-4.54).

During bivariate analysis, marital status was a significant factor for under-nutrition. Those who are unmarried were 2.7 times more likely to be undernourished than those who were married. Similarly, those PLHIV living single or at care homes were around three times more likely to be undernourished than those who resided with their families (Crude OR =3.45; 95% C.I, 1.68-7.05).

Household access to food is a key indicator for predicting under-nutrition. Of the 301 clients, 71 participants (23.6 percent) were found to have some food insecurity (denoted as levels 2, 3, or 4 on a scale of one to four). Households with food insecurity were more than twice as likely to be undernourished as those PLHIV with adequate access to food (Crude OR = 2.75; 95% C.I, 1.50-5.04).

Behavioral factors such as smoking and alcohol use were not found to be significantly associated with nutrition status. The association between various socio-demographic and behavioral factors and nutrition status is presented in Table 3.

**Table 3:** **Socioeconomic factors associated with the nutritional status**

**Variables**	**Categories**	**Underweight**	**Well-nourished**	**Crude OR (95% C.I.)**	**P-value**	**Adjusted OR (95% C.I.)**	**P-Value**
		**n(%) n = 60**	**n (%) n = 241**				
**Age**	≤36 years*	41 (25.8)	118 (74.2)	0.55 (0.28-1.09)	0.088		
	>36 years	19 (13.4)	123 (86.6)	1			
**Educational status**	Illiterate	22 (32.4)	46 (67.6)	**2.45 (1.33-4.54)**	**0.004**	**2.31 (1.10-4.82)**	**0.027**
	Literate	38 (16.3)	195 (83.7)	**1**			
**Gender**	Female	28 (23.9)	89 (76.1)	1.49 (0.85-2.64)	0.235		
	Male	32 (17.4)	152 (82.6)	1			
**Marital status**	Unmarried	13 (34.2)	25 (65.8)	**2.76 (1.36-5.59)**	0.005	2.14 (0.94-4.88)	0.701
	Separated/widowed/divorced	16 (32.0)	34 (68.0)	0.91 (0.31-2.22)	0.827	0.74 (0.24-2.29)	0.603
	Married	31 (14.6)	182 (85.4)	1			
**Ethnicity**	Dalits	6 (10.5)	51 (89.5)	2.42 (0.98-5.98)	0.054		
	Non- Dalits	54 (24.1)	190 (75.9)	1			
**Wealth quintile**	Middle class	20 (19.6)	82 (80.4)	1.30 (0.65-2.61)	0.458		
	Lower class	22 (22.2)	77 (77.8)	1.17 (0.54-2.31)	0.649		
	Upper class	18 (18.0)	82 (82.0)	1			
**Religion**	Hindu	44 (20.1)	175 (79.9)	0.76 (0.41-1.41)	0.391		
	Other than Hindu^#^	16 (19.5)	66 (79.1)	1			
**Residence**	Care homes/Single	16 (41.0)	23 (59.0)	**3.45 (1.69-7.05)**	**0.009**	**4.47 (1.79-11.15)**	**0.001**
	Family	44 (16.8)	218 (83.2)	**1**			
**Alcohol use**	No	44 (21.7)	159 (78.3)	0.71 (0.38-1.33)	0.278		
	Yes	16 (16.3)	82 (83.7)	1			
**Associated with PLHIV network**	No	43 (28.6)	150 (71.4)	1.54 (0.83-2.85)	0.175		
	Yes	17 (15.7)	91 (84.3)	1			
**Household food security**	Food insecure	24 (33.8)	47 (66.1)	**2.75 (1.50-5.05)**	**0.001**	**2.89 (1.39-5.95)**	**0.004**
	Food secure	36 (15.6)	194 (84.3)	1			
**Smoking habits**	No	41 (21.6)	149 (78.4)	0.75 (0.41-1.37)			
	Yes	19 (17.1)	92 (82.9)	1			
**HIV related factors**							
**Duration from diagnosis**	≤12 months	47 (24.7)	143 (75.3)	0.57 (0.32-1.03)	0.062		
	>12 months	13 (11.7)	98 (88.3)	1			
**ART**	Yes	50 (19.2)	211 (80.8)	0.71 (0.33-1.55)	0.391		
	No	10 (25.0)	30 (75.0)	1			
**CD4 count**	>350 cells/mm^3^	17 (10.4)	146 (89.6)	**0.26 (0.14-0.48)**	**0.000**	**0.26 (0.13-0.56)**	**0.000**
	≤350 cells/mm^3^	43 (31.2)	95 (68.8)	**1**			
**ART duration**	≤12 months	16 (21.1)	60 (78.9)	0.914 (0.46-1.81)	0.797		
	>12 months	44 (19.6)	181 (80.4)	1			
**TB infection**	Yes	17 (40.4)	25 (59.6)	**3.42 (1.70-6.86)**	**0.001**	**3.16 (1.43-7.01)**	**0.005**
	No	43 (16.6)	216 (83.4)	**1**			
**WHO clinical stage**	III/IV	41 (29.1)	100 (70.9)	**3.04 (1.67-5.55)**	**0.000**	**2.09 (1.04-4.21)**	**0.038**
	I/II	19 (11.9)	141 (88.1)	1			

1 = Reference Category.

*Mean value was used.

^#^Others include Muslim, Christian, Buddhist and Kirat.

Variables (education, age, gender, marital status, residence, OIs, WHO clinical stage, household food security and CD4 cell counts) are adjusted to minimize the effect of possible confounders in multivariate analysis.

### HIV-related variables and nutritional status

Various factors related to the HIV infection such as the duration from diagnosis, ART status, and duration on ART, presence of any OIs and WHO stage was integrated into study analyses. During bivariate analysis, no significant association was found between the time since diagnosis and nutritional status.

Differences between nutritional status and enrollment in the ART were assessed. A higher proportion of pre-ART PLHIV were undernourished than those on ART. Out of 40 PLHIV on pre-ART, ten were undernourished (25%). In comparison, out of 261 PLHIV on ART, 50 were undernourished (19.2.%). This association, however, was not statistically significant (Crude OR = 0.71; 95% C.I, 0.31-1.02) (Table 3).

### Multivariate analysis of associated factors

Those variables significantly related with under-nutrition (p-value <0.05) in bivariate analysis were further subjected into multivariate analysis using a logistic regression model. Only the variables found significant at 95% confidence interval in bivariate analysis were used for multivariate analysis. Binary logistic regression using the enter method was applied to get the final model, and the Hosmer and Lemeshow test was done to test goodness of fit. The model was found to be fit (p = 0.153 > 0.05). Multivariate analysis was performed to examine the independent effect of each of the significant independent variables in bivariate analysis with the effect of other variables remaining constant. In the multivariate analysis, the coefficient of determination (R^2^) was 0.67.

In bivariate analysis, marital status was found to be significantly associated with under-nutrition. However, this relation was not significant when subjected to multivariate analysis.

The association of literacy status with under-nutrition was found to be significant. Illiterate people were 2.3 times as likely to be undernourished as people who could read and write (Adjusted OR = 2.31; 95% C.I, 1.10-4.82).

PLHIVs’ residence with their family members, alone, or at care homes also determines their nutritional status. Residing with family members provides a protective effect against under-nutrition. Those participants who resided alone or in care homes were more than four times as likely to be undernourished than those living with their family members (Adjusted OR = 4.47; 95% C.I,1.79-11.14). Food security remains the prominent factor affecting nutritional status even when subjected to multivariate analysis (Adjusted OR = 2.88; 95% C.I, 1.39-5.95).

CD4 count was a significant factor associated with under-nutrition. PLHIVs with CD4 counts of 350cells/mm^3^ or more were 74% less likely to be undernourished than their counter parts (Adjusted OR = 0.26; 95% C.I, 0.12-0.55). Similarly, WHO clinical stages III and IV were found to be significant factors associated with under nutrition. PLHIVs in stage III and IV were more than twice as likely to be under-nourished than WHO stage I and II (adjusted OR = 2.09, 95% C.I, 1.04-4.21). Previous OIs with tuberculosis was also found to be the prominent risk factor for under nutrition (Adjusted OR = 3.16, 95% C.I, 1.42-7.0). The multivariate analysis is presented in Table 3.

### Health-related QoL of PLHIV

#### QoL descriptive statistics

QoL was measured using the WHOQolBREF [27]. Four domains capture different aspects of health-related QoL: physical health (including medical issues, pain, work capacity, and other aspects), psychological health (including negative and positive feelings, self-esteem, spirituality, and cognitive functions), social relationships (including social support and intimate relationships), and environment (including financial resources, safety, physical surroundings, and more).

#### Socio demographic factors and QoL

In the bivariate analysis of demographic variables and QoL scores, a significant difference in the QoL mean scores of male and female participants was noted. Males reported higher mean scores than females. Literate participants were also found to have higher QoL scores. Other demographic characteristics, such as marital status, religion and occupation, showed no significant differences in QoL scores.

Mean QoL scores were also found to be significantly higher among PLHIV with higher CD4 counts and those who were at WHO clinical stage I and II. The association, however, was not significant in the physical health and social domains.

The mean scores for all four different domains were found to be lower among the undernourished than those PLHIV having a BMI >18.5 kg/m^2^ and this difference was significant for psychological, social and environmental domains (Table 4).

**Table 4:** **Mean scores of different domains of the QoL**

**Variables**	**Categories**	**Physical domain ± SD**	**Psychological domain ± SD**	**Social domain ± SD**	**Environmental domain ± SD**
**Gender**	**Male**	48.8 ± 9.8	46.5 ± 10.8	43.8 ± 13.	44.5 ± 10.9
	**Female**	45.3 ± 8.3	41.7 ± 10.2	38.5 ± 12.3	40.2 ± 9.5
**Education**	**Illiterate**	45.8 ± 9.6	45.8 ± 10.6	43.0 ± 13.4	44.5 ± 10.7
	**Some education**	40.6 ± 7.8	40.6 ± 10.6	36.6 ± 11.3	37.3 ± 8.2
**CD4 count (cells/mm** ^**3**^ **)**	**<350**	46.4 ± 10.10	43.3 ± 11.4	41.0 ± 12.9	41.6 ± 11.0
	**≥350**	48.6 ± 8.5	46.2 ± 9.8	42.6 ± 13.4	44.3 ± 9.8
**WHO stage**	**I/II**	46.2 ± 8.0	41 ± 8.9	39 ± 9.3	40 ± 8.2
	**III/IV**	48.38 ± 8.4	46 ± 10.6	43 ± 14.5	44.9 ± 11.2
**BMI (Kg/m** ^**2**^ **)**	**<18.5**	45.41 ± 9.4	41.5 ± 11.7	36.06 ± 12.3	38.4 ± 9.4
	**≥18.5**	47.9 ± 10.6	45.4 ± 10.4	43.1 ± 13.0	43.9 ± 10.6

#### Correlation between QoL and nutritional status

A Spearmen Rank correlation test was carried out to observe if there was any correlation between nutritional status and QoL. A positive correlation was seen between weight and all four domains, as well as between BMI and all four domains. However, the correlation is weak (<0.3) throughout. Notably, height is also found to be significantly correlated with three domains (psychological, level of independence and environment) of QoL (Table 5).

**Table 5:** **Correlation between the nutritional status and QoL**

**Domains/Nutritional status**	**Physical**	**Psychological**	**Level of independence**	**Environment**
**Weight**	0.124*	0.236*	0.272*	0.262*
**Height**	0.059	0.148*	0.207*	0.164*
**BMI**	0.136*	0.154*	0.208*	0.215*

*Significant at 95% CI, P value <0.05.

## Discussion

### Nutritional status of PLHIV

The study estimates the rate of under-nutrition among PLHIVs and assesses correlations to other elements of their lives. The role of HIV infection on nutrition has been well-documented. Wasting is one of the most visible signs of malnutrition in patients who progress to AIDS [34]. Food insecurity, the condition of not having physical or economic access to sufficient food to meet dietary needs for a productive and healthy life [35], remains a common problem among PLHIV despite advances in their treatment and survival. However, the complex interactions of under-nutrition, food insecurity, QoL, and health status are not well-documented.

HIV affects the proportion of population already struggling with malnutrition and food security. In 2011, the Nepal Demographic and Health Survey (NDHS) reported that 18% of all adult females in Nepal were malnourished. The study suggests that HIV-infected women are more vulnerable to malnutrition, with 23 percent of female participants having BMI less than 18.5 kg/m^2^ [36].

According to the WHO, nutritional support should be fundamental to a comprehensive response to HIV and AIDS [37]. Poor nutritional status in PLHIV is associated with disease progression, increased morbidity, and reduced survival, even when ART is available [38]. A study in the United States found that micronutrient supplements significantly increased CD4 count among PLHIV [39], and studies among HIV-infected adults in Haiti, Kenya, Malawi and Zambia have demonstrated significant positive effects of macronutrient supplementation on adherence to antiretroviral medication, weight gain and CD4 counts [38-42]. Similarly, in food-insecure settings, food support programs (increasing total calories) are often required in addition to nutrition support (increasing specific micronutrients) to optimize health outcomes in PLHIV [38]. This supports the idea of nutritional support to PLHIVs in countries (like Nepal) where food insecurity is endemic.

### Factors associated with under nutrition among PLHIV

Various socio-demographic and HIV-related factors determine the nutritional status of PLHIVs. Education status was significantly associated with nutritional status, in that illiterate people were more likely to be undernourished. Previous studies of underlying factors in under-nutrition among the illiterate demonstrate that differences may include their dietary pattern, their understanding on the disease processes, and ART adherence [43]. A study carried out in Ethiopia in 2013 indicates that occupation and economic status might account for the vulnerability of illiterate people to under nutrition [44]. In this study, no significant association was seen between under-nutrition and socioeconomic status.

Independent of all other variables, WHO clinical stages III and IV have significant effects on the likelihood of malnutrition development. Malnutrition is usually encountered at the advanced phase of HIV infection. An anthropometric measurement like BMI is lower in symptomatic patients as classified by WHO HIV disease stages [45]. Since disease status could modulate the immunological responses to HIV infection over time [44], further research with longitudinal design can be helpful in assessing the effect of malnutrition on HIV infection progression.

It was found that an OIs (tuberculosis) was an independent risk factor for under nutrition. Similar other studies support the findings that TB is associated with under nutrition [46-49]. The HIV-induced immune impairment and heightened subsequent risk of OIs can worsen nutritional status [50]. This exemplifies the importance of managing patients with OIs promptly.

Regarding CD4 count, we found that PLHIV with more than 350 cells/mm^3^ were more well-nourished than PLHIV with less than or equal to 350 cells/mm^3^. Since this is a cross-sectional study, some longitudinal study can better explain the relationship between CD4 count and nutritional status. The same is true for understanding the relationship between OIs and under nutrition.

### Correlation between nutritional status and health related QoL

There are very little evidence linking nutritional status and health related QoL. In our study all three parameters (height, weight, and BMI) were found to be significantly associated with the domains of QoL. A cross-sectional study carried out at West Bengal, India, among HIV-positive women in 2012 similarly showed that QoL scores were correlated positively with all the parameters of nutritional status [12]. The correlation of height and QoL may indicate that lifelong undernourishment resulting in stunting, rather than current HIV status, may correlate with lower levels of QoL. However, further investigation is necessary to determine this.

### Limitations

The cross-sectional nature of the study limits the investigation into causal relationship between determinants and outcomes of interest (malnutrition). Likewise, further comparative study between HIV-infected and non-HIV infected persons could explore additional nutrition and food security risk factors. The study, like others, is subject to some systematic errors, including measurement error, recall bias for self-reported components, and incomplete medical records. These are the key limitations. Additionally, self-reported HRQoL measures may feature important confounds, in that some aspects of HRQoL may also be affected by factors in a study participants life unrelated to nutrition status or HIV and AIDS that nevertheless affect their answers.

## Conclusions

In our findings, one in every five PLHIV is undernourished. Nutritional status was found to be positively correlated with QoL. Although further longitudinal research will be helpful in determining the role of nutritional status in QoL, the results of this study suggest that the existing care options for PLHIV do not appear to fully address this issue. Nutritional support should form a fundamental part of the response to HIV and AIDS in Nepal, including more efforts toward providing nutritional counseling, support, and encouragement to clients. Our results also make a case for a PLHIV-specific food security program, additional to ones targeting HIV-negative food-insecure populations, to ensure this especially vulnerable population receives adequate support.
